# Defending rice crop from blast disease in the context of climate change for food security in Nepal

**DOI:** 10.3389/fpls.2025.1511945

**Published:** 2025-06-25

**Authors:** Ram Bahadur Khadka, Hira Kaji Manandhar, Sundar Shrestha, Basistha Acharya, Pratiksha Sharma, Suraj Baidya, Van Schepler Luu, Krishna Dev Joshi

**Affiliations:** ^1^ Nepal Agricultural Research Council, National Plant Pathology Research Centre, Lalitpur, Nepal; ^2^ Nepal Plant Disease and Agro Associates, Kathmandu, Nepal; ^3^ Nepal Agricultural Research Council, National Rice Research Program, Dhanusha, Nepal; ^4^ International Rice Research Institute, Rice Breeding Innovation Platform, Los Baños, Laguna, Philippines

**Keywords:** climate change, durable blast resistance, early detection, food security, Nepal, blast pathogen diversity, rice

## Abstract

Blast, caused by *Pyricularia oryzae* (teleomorph *Magnaporthe oryzae*), is one of the most devastating diseases in rice, causing 10-30% yield losses and threatening Nepal’s food and nutritional security. The Himalayan foothills are hotspots for blast fungus diversity, leading to the rapid emergence of pathotypes that overcome resistance in mega rice varieties. In 2022, a neck blast epidemic devastated 5,000 hectares of Hardinath-1, a dry winter/spring rice variety in Chitwan, causing nearly 100% yield loss. The changing climate, especially during panicle initiation stages, has become more favourable for neck blast development. We reviewed 40 years of research and development on rice blast in Nepal, analysing historical weather patterns and mapping the incidence and severity of the disease across the country based on empirical observations and field experiments. Using historical data on rice blast incidence and climate information, we show that rice blast pressure is increasing intensively and changing weather patterns are becoming more favourable for rice blast epidemics. We identify emerging issues in rice blast and propose integrated strategies for effective management in Nepal. Key approaches include developing durable blast-resistant and climate-resilient rice varieties using molecular markers and genomic tools and speed breeding, forecasting disease and pathotype emergence, and combining these with careful use of modern fungicides, plant defence activators, and biological control. Additionally, adjusting planting times, managing weeds, optimising agronomic practices, and ensuring proper water and nutrient management are essential for sustainable blast management.

## Introduction

Rice (Oryza sativa) is a staple crop globally, including in Nepal, where it contributes significantly to food security. It accounts for about 75% of total cereal consumption, over 30% of caloric intake, and 23% of protein intake (CBS, 2016). In Nepal, rice is cultivated in two seasons: wet-season rice (main crop) and dry winter-season rice, popularly known as *Chaite rice* (spring rice). Due to inadequate domestic production, the country heavily relies on imports to meet its growing demand (Gauchan et al., 2022).

Rice blast, caused by the fungus *Pyricularia oryzae* (teleomorph *Magnaporthe oryzae*), is among the most devastating diseases of rice worldwide (Zeigler et al., 1994). This disease threatens both lowland and upland rice production around the world (Kuyek, 2000; Kato, 2001; Talbot, 2003; Dean et al., 2005). This disease also impacts other cereal crops, including wheat, barley, and millets. It infects all growth stages of rice, including leaf, collar, neck, and panicle, leading to reduced yield and quality (Khan et al., 2014). The disease is particularly severe in upland, rainfed rice fields (Tastra et al., 1987). In Nepal, rice blast was first recorded in 1964 from Thimi, Bhaktapur (Bhatta, 1966) and remains a significant challenge across all rice-growing altitudes (100 to 3000 m). Without effective management, rice blast can cause total crop failure, posing critical risks to Nepal’s food security and economy (Manandhar, 2017; Agbowuro et al., 2020), highlighting the urgency of addressing this disease with a national priority.

The rice blast pathogen is a filamentous heterothallic ascomycete. It initially grows as a biotroph in rice leaves, before transitioning to a hemi-biotrophic lifestyle infecting, and multiplying within living plant cells before killing them and spreading to neighbouring cells (Wilson and Talbot, 2009). This fungus produces toxins such as pyricularin and α-picolinic acid, with tenuazonic acid contributing to necrotic lesions on infected leaves (Umetsu et al., 1974). Under favourable conditions, the pathogen completes its life cycle within a week. A single lesion can release hundreds of spores nightly for over 20 days, with secondary infections primarily caused by conidia dispersed through air currents (Kato, 2001; Sopialena and Palupi, 2017). Optimal conditions for rice blast development include high humidity, leaf wetness, and temperatures between 17 to 28°C (Greer and Webster, 2001; Muñoz, 2008). Infection during the vegetative stage reduces light absorption and grain quality (Bastiaans et al., 1994; Bregaglio et al., 2017).

Blast epidemic causes complete loss of seedlings (Chaudhary et al., 1994), yield reduction by neck blast infection is twice as severe as leaf blast (Hwang et al., 1987). Neck blast, the most destructive phase, can result in complete crop loss if it occurs before the milking stage (Goto, 1965; Zhu et al., 2005).

Rice blast spreads from plant to plant through both seeds and air. In Nepal’s Terai and inner Terai regions, high soil temperatures in June suppress seed-borne inoculum from causing infection (Manandhar, 1998c). However, in temperate regions, pathogen overwinters on straw, seeds, and alternative hosts such as *Eleusine coracana*, *E. indica*, *Panicum* spp., *Setaria* spp., serve as primary sources of infection (Zeigler et al., 1994). While the *Pyricularia* genus parasitizes over 50 hosts, its strains typically have narrow host ranges, and cross-infection is rare (Zeigler et al., 1994).

Nepal, recognised as a biodiversity hotspot for the rice blast pathogen (Saleh et al., 2014), prompting numerous studies on its biology, ecology, and pathotype diversity. However, much of its research is fragmented and not easily accessible online. This research consolidates existing knowledge on rice blast, including its biology, ecology, potential impacts of climate change, and disease management strategies. It also highlights advances in global research, such as gene editing and genomic selection, and speed breeding, which offer promising avenues for accelerating varietal development and improving disease management. By exploring these technologies, this research aims to address key challenges to ensure food and nutritional security in Nepal.

The urgency of addressing the rice blast in Nepal is critical due to its severe impact on food security and the economy. This fungal disease, which causes up to 30% yield losses, threatens both lowland and upland rice production. In 2022, a neck blast epidemic wiped out 100% of the spring rice crop in Chitwan, underscoring the vulnerability of Nepal’s rice supply. Climate change is exacerbating the situation, increasing the frequency of outbreaks. Effective management, including developing resistant varieties and improved farming practices, is essential to protect Nepal’s rice production and ensure long-term food security.

## Methodology

Neck blast and leaf blast surveillance were conducted at Khumaltar, Lalitpur (1300 m), a mid-hill valley on a standard rice blast screening nursery in 2021 and 2022 (NPPRC, 2022, 2023; NRRP, 2022, 2023). The visual detection and assessment of blast infection was scored on a 0–9 scale following the Standard Evaluation System (SES) for rice developed by the International Rice Research Institute (IRRI) (IRRI, 2013). In addition, leaf blast severity data collected from the National Rice Blast Screening Nurseries in 2019, 2020, and 2021 by the Rice Research Network of NARC were gathered from locations across the Terai region: Tarahara; Sunsari (eastern, 139 m), Hardinath; Janakpur (central, 75.76 m), and Khajura; Banke (western, 165 m) (NPPRC, 2020, 2021). These scores were converted to percent severity using mid-values, and mean scores were presented in a heatmap using R function Heatmap (R Core Team, 2024).

Weather data from 1980 to 2023 on daily precipitation, relative humidity, and maximum temperature were obtained from the Department of Hydrology and Meteorology, Government of Nepal (DHM, 2024). Data for dry winter season rice (April-May flowering) and wet season rice (September-October flowering) were extracted. The correlation of the climatic conditions and rice blast incidence and severity were interpreted.

Twenty-five districts representing major rice-producing domains across the country were selected in alignment with NARC’s blast screening sites to gather quality data. Disease severity data from the National Rice Blast Screening Network were reviewed, and expert knowledge and experience were used to validate risk scores for neck and leaf blast epidemics assigned to each site.

Leaf and neck blast severity were recorded using IRRI’s Standard Evaluation System (SES) with a 0–9 scale (IRRI, 2013). Scores were categorised into three groups: 1 = 0-3 (low vulnerable), 2 = 5-7 (medium/moderately vulnerable), and 3 = 9 (highly vulnerable), then plotted on Nepal’s physiographic map using ArcGIS 10.3.

To compile a comprehensive overview of current knowledge on rice blast in Nepal, a structured methodology was used to select articles for review. Queries were conducted in key academic databases, including Web of Science, Scopus, Google Scholar, and NARC e-library using terms such as “rice blast in Nepal”, “*Pyricularia oryzae* in Nepal,” and “*Magnaporthe oryzae* in Nepal.” Results were filtered based on title, abstract, and keywords, initially removing duplicates and manuscripts from predatory journals. Subsequently, the full texts, keywords, and titles of the remaining articles were carefully assessed. Around 100 research articles including grey literature and reports were summarised in this review.

## Results and discussion

### Evidence for increasing rice yield loss due to rice blast in Nepal

Rice blast, particularly neck blast, is increasingly prevalent and severe in Nepal, leading to significant economic losses annually. A field experiment at Khumaltar (mid-hill valley) showed that 1% increase in neck blast led to 21 to 51 kg/ha grain yield reductions in a highly susceptible cultivar “Sankharika” (Manandhar et al., 1985), and at (Terai) 38.5 to 76.1 kg/ha grain yield reductions in Masuli and Radha-17 cultivars, respectively (Chaudhary, 1999). Epidemics of blast in the rice nursery have been reported in the 1980s, resulting in a complete loss of the seedling (Adhikari and Shrestha, 1986; Pradhanang, 1988). Earlier research also highlighted the challenge of blast pathogens overcoming resistance mechanisms of resistant varieties and in certain cases very quickly. For example, the two blast-resistant varieties Himali and Khumal-3 became susceptible to neck blast in farmers’ fields within 3–5 years of their release (Thapa and Manandhar, 1985). Similarly, Lekali Dhan-1, another leaf blast resistant variety being heavily infected by leaf blast in Dolakha after 9 years of release (NPBGRC, 2024; NPPRC, 2024). Several introduced rice varieties recommended for diverse agroecological regions frequently experience the rapid loss of blast resistance. It is primarily because all of these were bred for high yield with a combination of all the desirable traits including blast tolerance not specifically bred as varieties with durable resistance to blast.

For example, Himali was bred using Cica-4 and Kalu for high yield and adaptability for high-altitude areas (Joshi et al., 2014a). Precise genetic information on blast resistance of both the parents of Himali on (Cica 4 and Kalu-a Nepali native variety) is limited. Cica-4 was developed in Latin America during the mid-20th century for high yield and adaptability to various environmental conditions. Given the prevalence of blast disease in rice-growing regions, it is reasonable to infer that Himali may possess some level of blast resistance inherited from its parentage.

Khumal-3 was bred using China1039/IR580 (Joshi et al., 2014b). While specific details about blast resistance genes in China 1039 are not widely documented. While IR580, an IRRI breeding line, is often used in breeding for traits like drought resistance and high yield, it is also likely to carry blast resistance genes. In fact, many IRRI varieties, especially those in the IR series, have been bred with disease-resistant traits. IR580 may carry genes like Pi-k, Pi-ta, or other minor resistance genes known to be effective against certain pathotypes of rice blast. Blast resistance in Khumal-3 likely comes from both China 1039 and IR580, which may carry genes like Pi-ta, Pi-z, or Pi-k.

However, as Himalayan foothills of Nepal are hotspots for blast, it is possible that the resistance of introduced rice varieties such as Himali, Khumal-3 and Lekali Dhan-1 could break down more quickly, especially when new, more virulent strains of the pathogen emerge.

**Figure 1:**
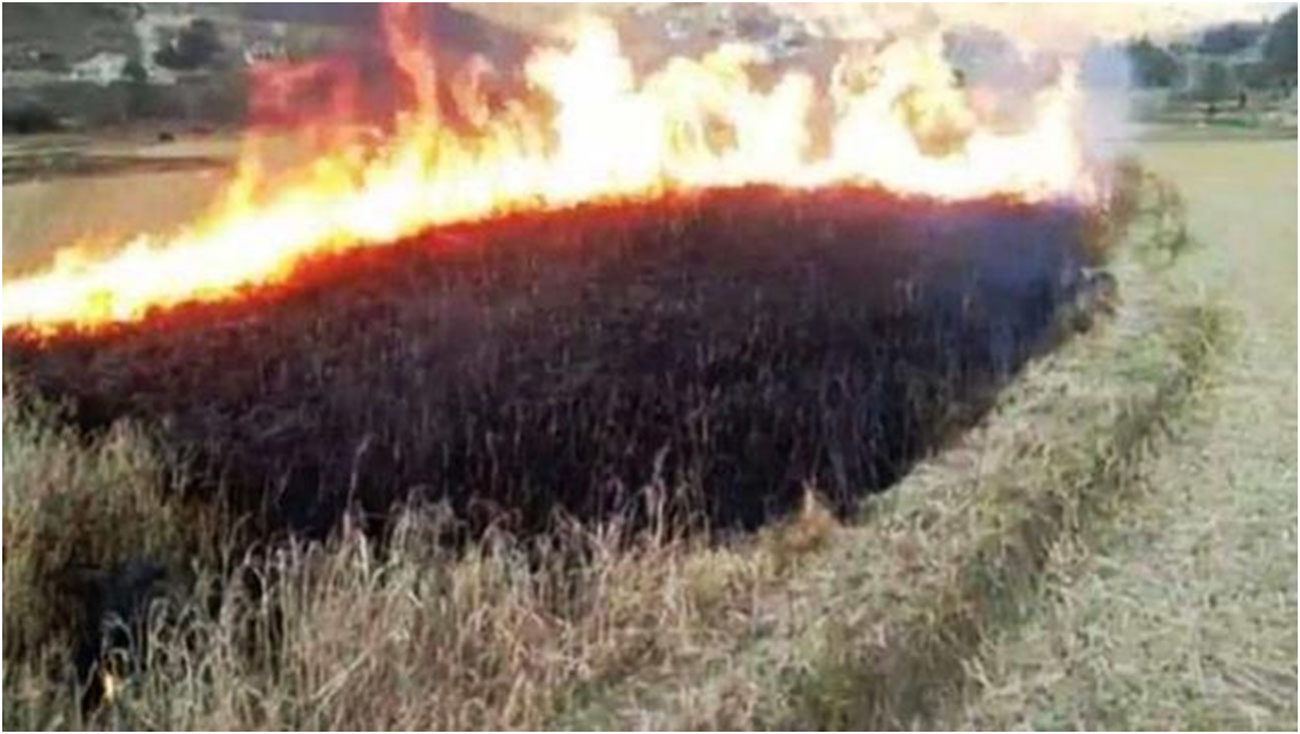
Farmers burning rice after severe incidence of neck blast in Marshi rice in Tatopani village in Jumla valley of Nepal. (Figure adapted from Nayapatrika daily) picture taken at 4 November, 2022.

In high hill and mountain areas, high humidity and dew during the panicle initiation stage, combined with cooler temperatures, result in prolonged drying times and increased neck blast incidence. For instance, in Jumla valley (3000 m), in case of blast epidemics farmers resort to burning the blast-susceptible Jumli Marshi crops without harvesting. In the Terai, inner Terai, *Besi*, and *Tar* regions during June-July, high rainfall and cloudy weather create conditions that promote fungal spore germination, leading to higher neck blast in early-season rice. For instance, Paudel (2020) reported a severe neck blast infestation on the Hardinath-1 rice variety in Chitwan Valley, leading to a 100% yield loss (Chhetri, 2015, **Figure 1**).

Prolonged monsoon (after mid-September) in hilly areas and river basins in main-season rice can cause severe neck blast, potentially resulting in over 50% yield loss. In 2022, substantial losses due to neck blast were observed in central (Dolakha, Kabre, Nuwakot) and western (Tanahu, Lamjung, Kaski) hilly regions (NPPRC, 2024).

(**Figure 2**).

**Figure 2:**
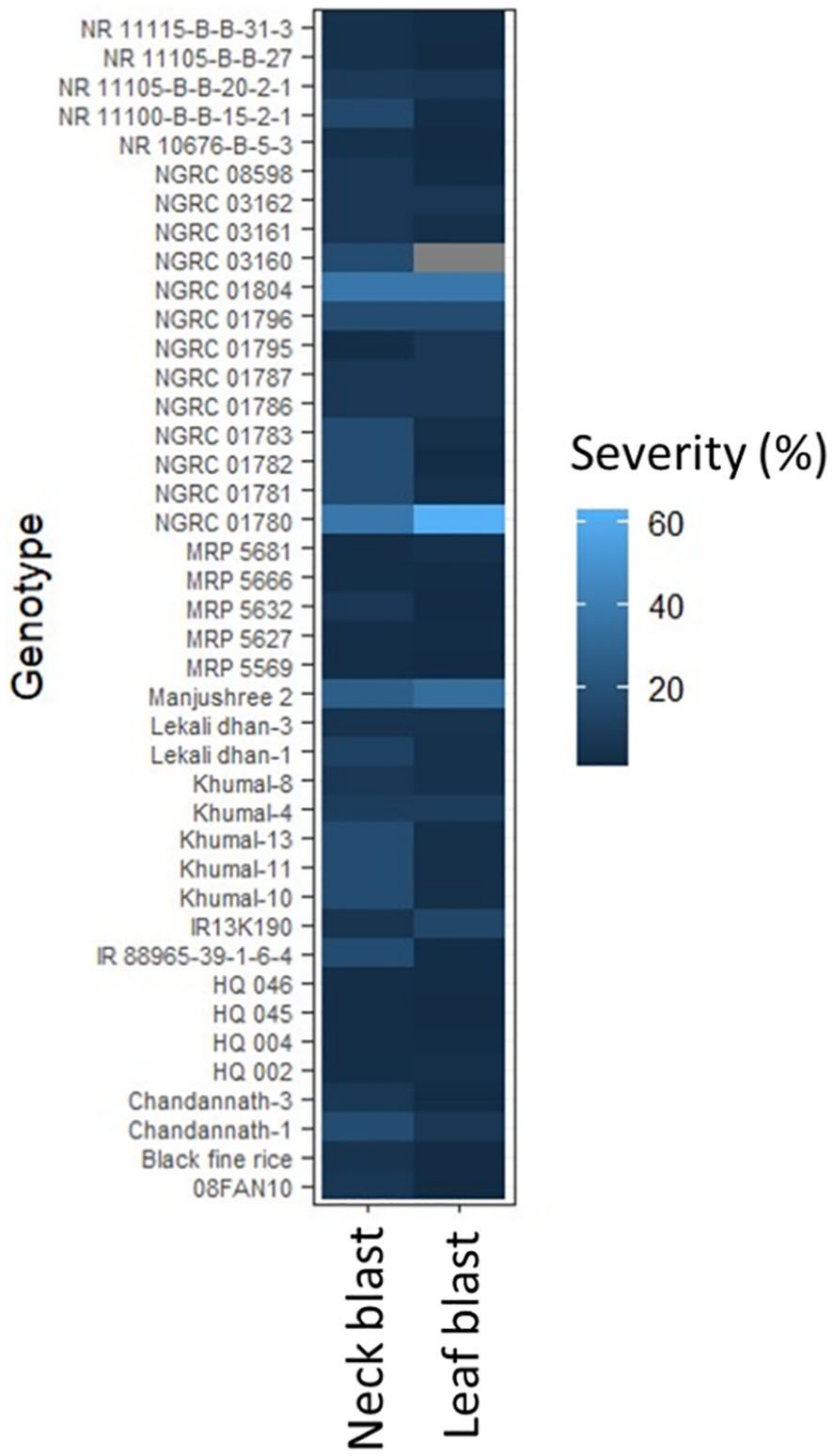
Reaction of rice genotypes with leaf blast and neck blast in 2021 and 2022 at central hills of Nepal.

**Figure 3:**
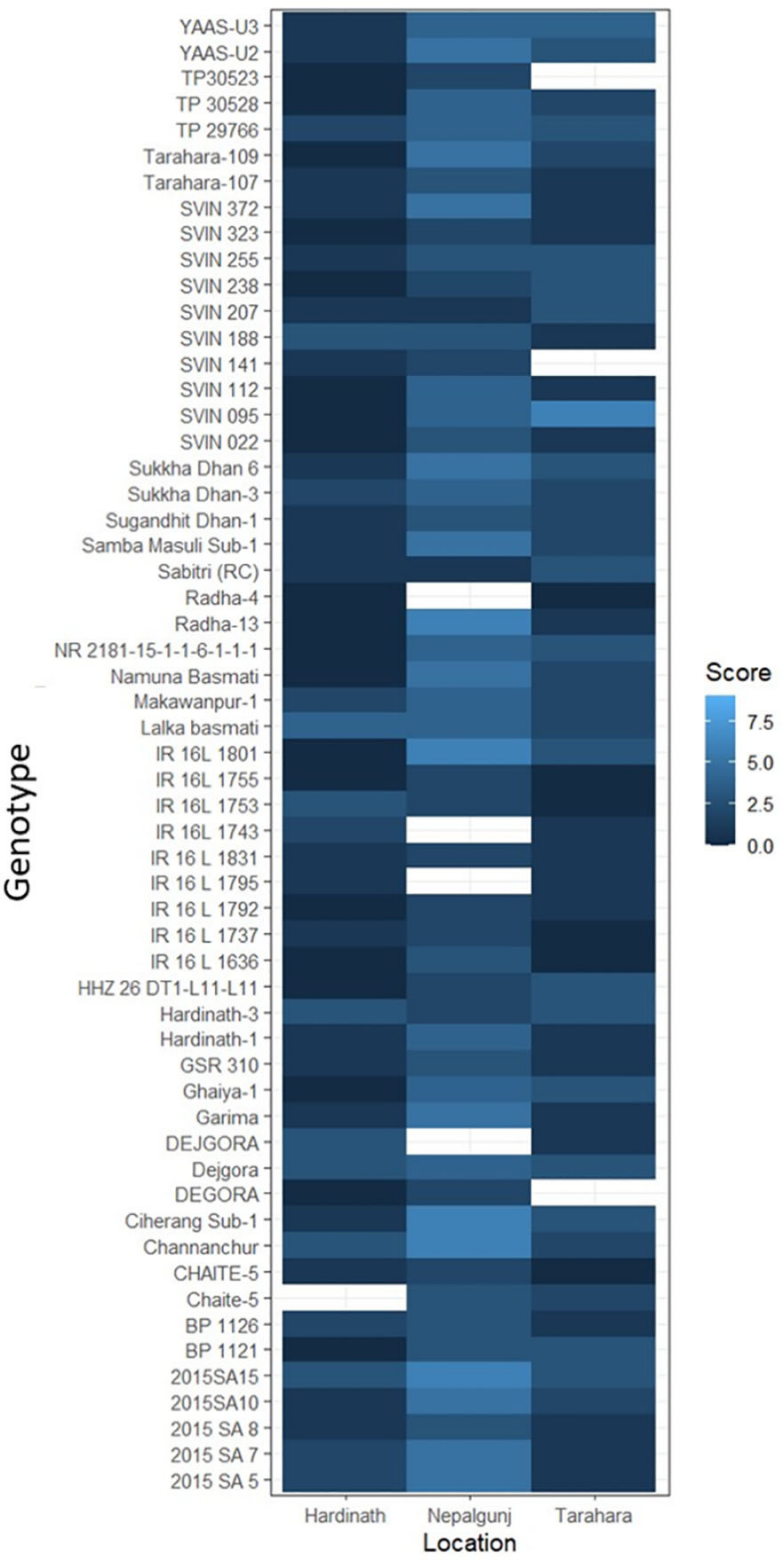
The reaction of rice genotypes with leaf blast in 2019, 2020 and 2021 at the eastern plain (Tarhara), central plain (Hardinath) and western plain (Nepalgunj) regions of Nepal.

Late planting of rice in rainfed conditions due to inadequate or irregular rainfall is common in Nepal, this coupled with subsequent high rainfall during flowering in hilly areas of Nepal fosters conditions ideal for neck blast, causing annual devastation (**Supplementary Figure 1**; **Figure 3**). A farmer-preferred short-duration rice variety, Chaite 5, released by NARC in 2018 encountered heavy infection of neck blast in 2023 in Chitwan valley, which raised concerns among growers for its future cultivation (Dhan Bahadur Thapa, personnel communication, 2024).

**Figure 4:**
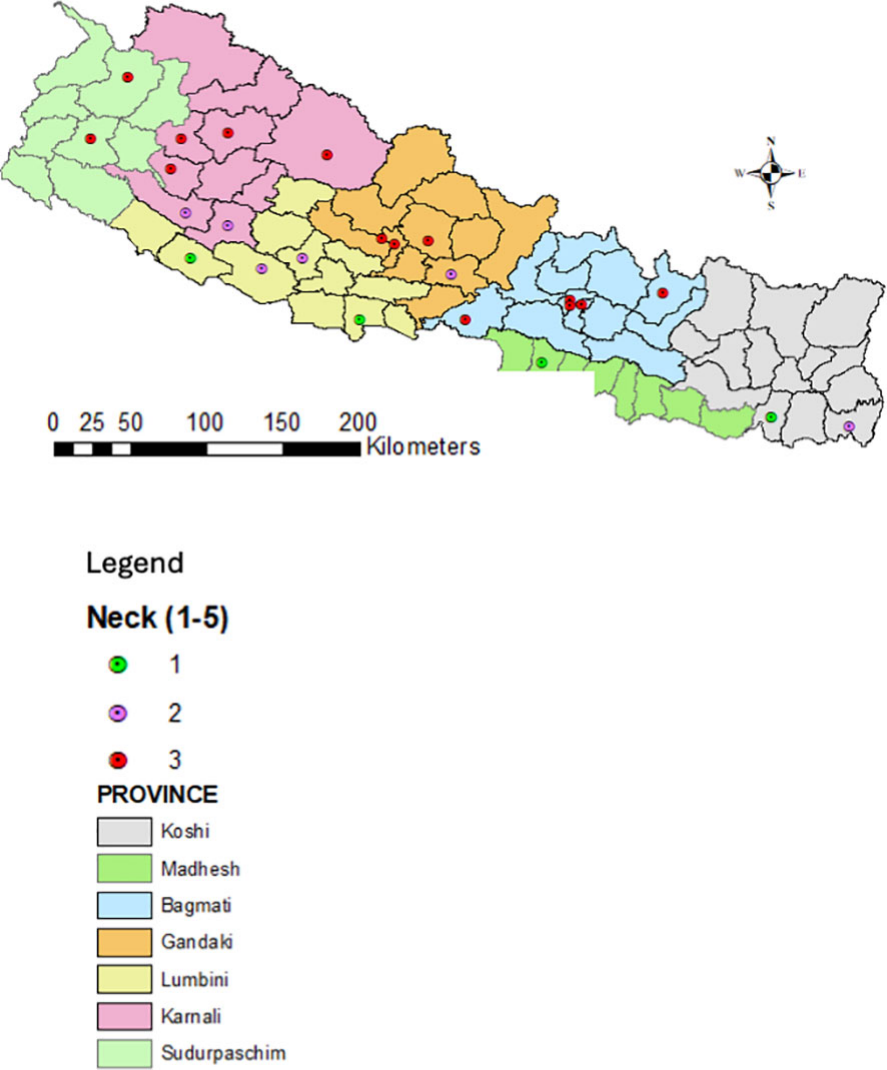
Severity of the rice neck blast across different geographical domains of the country.

**Figure 5:**
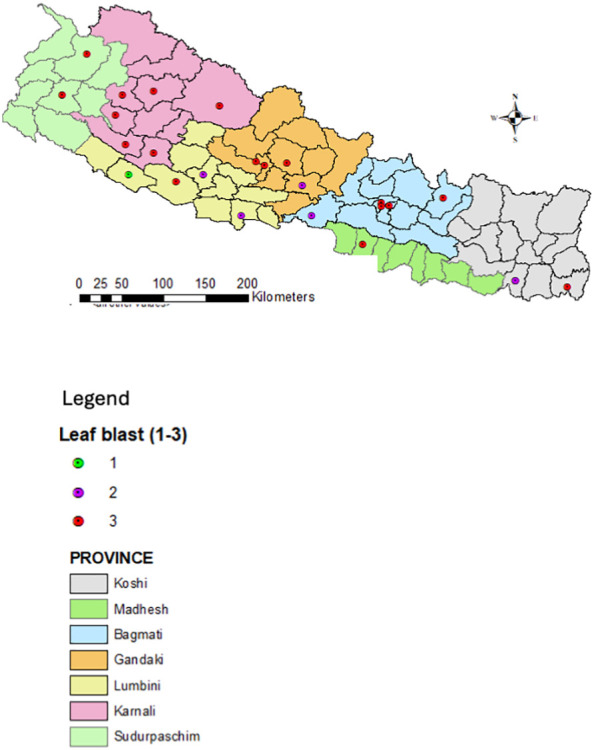
Severity of rice leaf blast in different geographical domains of the country.

The eastern Terai of Nepal has higher humidity throughout the year due to its proximity to water bodies, dense vegetation, continuous monsoon rains, and its low-lying topography. The western Terai, while also humid, experiences slightly lower humidity, especially in the dry season, due to its distance from the monsoon winds and more arid conditions. These factors contribute to high blast incidence in east Terai compared to west (**Figures 4**, **5**).

### The diversity of Nepal’s agroecological conditions provides habitats suitable for rice blast

Different physiographic regions of Nepal have varying soil types, altitudes, moisture regimes, and cropping systems, resulting in diverse agroecological niches. Nepal’s diverse topography and agroecological conditions provide a range of microclimates and habitats suitable for different rice varieties which can shelter the diversity of the blast fungus. This complex landscape, climatic diversity along with the diversity of rice varieties and human activities can offer a fertile environment for the evolution, emergence and adaptation of various rice blast races in Nepal.

In low-altitude areas of Nepal (up to 1,200 m), rice is cultivated in two cropping seasons: wet season (June/July to November/December) and dry season (January to June/July). These two seasons overlap for at least one month, which provides the green-bridge for the movement of blast fungus from one cropping season to the next. Green-bridge facilitates pathogen transmission, building up inoculum to trigger disease epidemics. Voluntary rice plants and barnyardgrass (*Echinochloa crus-galli*) act as inoculum sources, creating a robust green-bridge that drives rice blast epidemics.

The interaction between two rice cropping seasons with various varieties can result in the selection of new, more virulent strains capable of overcoming the varietal resistance.

Various pathotypes and races of rice blast have been identified in Nepal, with different regions hosting distinct strains, some of which match those found in other parts of Asia. Nepal is recognised as one of the important centres for rice blast diversity (Saleh et al., 2014). Chaudhary et al. (2004) reported 15 pathotypes. Thapa and Manandhar (1985) reported the race group ‘IC’ in Khumaltar (mid hill valley), and ‘IA’ in Parwanipur (Terai). Interestingly, the race group of Khumaltar matches with eastern Asian races and races from the Terai region is one of the most virulent races in the globe (Thapa and Manandhar, 1985). Although previous studies indicated no evidence of sexual reproduction in blast fungus in the country and only mating type A is present (Yaegashi and Yamada, 1986. However, more recent studies indicate the presence of both mating types in Nepal but still there is no evidence of sexual reproduction (Saleh et al., 2014), this might be due to chance of escape due to low numbers of sample coverage. Extreme climatic variability, rice diversity, a continuous green bridge, and the presence of both mating types provide a strong foundation for high pathotype diversity in Nepal. Therefore, detailed studies profiling pathotype diversity across all microclimatic domains are essential to guide rice blast-resistant breeding strategies in the country.

### Climate change worsen the impact of rice blast in Nepal

Nepal’s climate ranges from tropical in the southern plains to alpine in the high Himalayas, encompassing various intermediate climatic zones. These climatic variations can influence the prevalence and severity of rice blast. Certain areas such as Mulpani in Kathmandu that have higher humidity (average humidity of around 70-95% in July-October) and suitable temperature conditions (ranging from 20 to 35°C) provide hot spots for rice blast nurseries (NPPRC, 2024). However, as climate changes, the spread of rice blast may change geographically, areas once unsuitable for the disease may become favourable, while traditional hotspots may see alterations in disease prevalence due to local climate changes. These shifts affect both local and global rice production (Coakley et al., 1999).

Climate change is expected to exacerbate the spread and severity of the disease, as alterations in temperature, humidity, and rainfall patterns create more favourable conditions for blast outbreaks and subsequent crop losses. Since rice blast fungus infects during periods of high humidity and prolonged leaf wetness at moderate temperatures (20-30°C) (Kirtphaiboon et al., 2021), changes in precipitation can affect leaf wetness duration and humidity levels, crucial for disease progression while prolonged leaf wetness and high humidity can enhance the germination of fungal spores and aid in disease transmission. In addition, temperature, precipitation, and dew also influence various stages of the disease cycle, including spore attachment, viability, germination rates, and penetration potential (Asibi et al., 2019). Furthermore, climate change-induced stress such as heat, drought, and cold waves can also significantly impact rice blast disease by reducing host resistance and influencing rice growth and development. An analysis of 50 years of weather data from Nepal’s central mid-hills indicates rising humidity and rainfall during the panicle initiation and flowering stages, likely driven by climate change, which has intensified disease severity and frequency (**Supplementary Figures 1**–**3**). Notably, shifting rainfall patterns toward late September and early October (Sijapati et al., 2014) align with panicle initiation, leading to a significant increase in neck blast incidence.

Climate change can impact how rice plants interact with rice blast pathogens on a molecular level. Temperature and humidity variations can affect gene expression related to plant defence and pathogen virulence, potentially shifting the balance between host resistance and pathogen aggressiveness. This could result in the emergence of new pathogen strains better suited to the changing climate, potentially overcoming previously resistant rice varieties (Devanna et al., 2022).

Rice blast is significantly influenced by temperature changes (Manibhushanrao and Day, 1972; Mao et al., 1997). Field studies show that 28°C–30°C suppresses outbreaks, while 24°C–26°C promotes them (Padmanabhan, 1965; Katsantonis et al., 2017). Temperature also regulates R gene-mediated resistance, with high temperatures compromising Pi54 resistance but enhancing Pib-mediated resistance (Madhusudhan et al., 2019; Wang et al., 2001). Qiu et al. (2022), identified decreased JA biosynthesis and signalling under warm conditions as the cause of reduced basal resistance, leading to downregulation of genes like *OsCEBiP* and increased rice susceptibility.

Humidity plays a critical role in rice blast epidemics. Studies done by Qiu et al. (2022) reveals that high ambient humidity promotes rice blast development by enhancing *M. oryzae* virulence, including conidial germination and appressorium formation. RNA sequencing and ethylene assessments show that high humidity suppresses ethylene accumulation and signalling activation induced by *M. oryzae*. High ambient humidity increases *M. oryzae* virulence and reduces rice basal resistance by impairing ethylene biosynthesis and signalling, key components of humidity-modulated defence mechanisms. Qiu et al. (2022) further demonstrated that ethylene signalling plays a pivotal role in the interaction between humidity signals and rice immunity. Under high humidity, *OsEIN2* and *OsEIL1* expression is reduced in response to *M. oryzae*, facilitating the development of rice blast.

### Slow seed replacement rate contributed to increased rice blast disease in Nepal

Most of the farmers in Nepal grow the seed saved from their own farms and exchange the seeds with neighbouring farmers. Findings from Sapkota et al. (2017) revealed that the rice seed replacement rate for improved rice varieties was only 8.7%, which is quite low. Due to the predominance of smallholder farmers 80 to 90% of planting materials come from farmers’ seed systems in South Asia and Sub-Saharan Africa (GRAIN, 2007) and farm-saved seeds may be carriers for blast fungus. Studies show that the old and obsolete varieties persist in the rice production system of Nepal (Gauchan and Pandey, 2012; Witcombe et al., 2016; Joshi et al., 2023) that can contribute to disease epidemics. A recent study revealed the predominance of old and obsolete rice varieties in Nepal, in some cases, Nepalese farmers were unwillingly growing nearly 50-years old Indian rice varieties, like Hema (released in India in 1974) and Moti (released in India in 1988), falsely marketed as “new varieties” by Agrovets (Joshi et al., 2023) to boost seed sales. These varieties, not endorsed by Nepal’s Seed Quality Control Center (SQCC), were being sold without SQCC’s knowledge. Even more concerning is SQCC registered Sarju-52 (released in India in 1979) and Sona Mahsuri (released in India in 1982) in 2019 (SQCC, 2023), despite their susceptibility to various pests and diseases. Continuous planting of susceptible rice varieties in monoculture can increase blast prevalence (Mustafa et al., 2019) due to the ideal environment it creates for disease spread. Using old rice varieties also boosts disease pressure. Lack of genetic diversity makes it easier for the fungus to overcome resistance mechanisms.

### Changing rice farming practices and their influence on rice blast in Nepal

Crop management practices significantly affect the occurrence of blast disease. Despite advancements in agricultural technologies, Nepali farmers have been slow to adopt modern practices due to limited awareness, training, and extension services. This results in a continued reliance on inefficient traditional methods (Dhital, 2017). The small and fragmented land holdings further hinder the implementation of modern crop management techniques, reducing overall productivity (Adhikari et al., 2017).

Historically, rice planting was aligned with the monsoon season, which helped manage disease risks (FAO, 2019). Rice cultivation involves the widespread use of organic inputs such as farmyard manure (FYM), compost, and green manuring, along with a range of indigenous approaches to maintain and enhance soil fertility. Weeding was traditionally done manually (**Table 1**). However, the limited practice of split nitrogen application affects disease dynamics (IRRI, 2020; Karki et al., 2019). Improved nitrogen (N) management plays a critical role in reducing rice blast (*Magnaporthe oryzae*) by mitigating factors that promote disease susceptibility. Excessive nitrogen application leads to lush growth and increased humidity, creating conditions favorable for the pathogen (Fagard et al., 2014). Nitrate nitrogen supply influences rice resistance to bacterial leaf blight (Liu et al., 2024). Leaf blast was significantly more severe on the susceptible and very susceptible cultivars when N fertilizers was applied as a single application at preflood than in the split application treatment (Long et al., 2000). Enhancing nitrogen use efficiency (NUE) ensures effective nitrogen utilization, boosting plant defence mechanisms. Nitrate-based fertilizers are less conducive to blast development than ammonium-based ones (Ballini et al., 2013). Biofertilizers further enhance plant resistance due to activation of plant defence systems by microbial colonization in rhizospheres and phyllospheres (Bhattacharjee and Dey, 2014).

**Table 1:** Comparative analysis of rice blast management strategies used in Nepal and other rice growing countries.

Particulars	Nepal’s Context	Global Comparison	Reference
Agroecology and Habitat	Diverse agroecology with conducive microclimates for rice blast, especially during the monsoon	Similar conditions in India, China, and Indonesia, but Japan and South Korea mitigate risks using precision farming and disease support systems.	Sah et al. (2020); Singh et al. (2018)
Genetic and Varietal Diversity	Rich genetic diversity with traditional landraces, but slow varietal replacement and vulnerability to new blast strains.	Countries like China and the Philippines use molecular breeding to develop resistant varieties. Japan employs varietal diversification to reduce risks.	Joshi et al. (2017); Yang et al. (2020)
Farming Systems	Subsistence farming dominates, with limited adoption of knowledge-intensive practices. Weak technical knowledge transfer.	Industrial systems (e.g., Thailand, USA) use precision agronomy and ICT tools for monitoring. India is transitioning slowly to ICT-based advisory systems.	Neupane et al. (2019); Fujita et al. (2017)
Climate Impacts and Planting Time	Shifting planting times due to monsoon variability expose crops to rice blast during critical growth stages	China and Bangladesh mitigate risks through forecasting tools and staggered planting schedules. Nepal lacks regional advisories for planting.	Adhikari et al. (2022); Hossain et al. (2020)
Integrated Practices	Limited adoption of crop rotation, seed treatment, and biocontrol agents. Heavy reliance on farm-saved seeds and inconsistent and haphazard fungicide use.	India and Vietnam promote integrated practices. Brazil emphasizes biocontrol agents and fungicides. Japan integrates biological and chemical controls effectively.	Ghimire et al. (2020); Sharma et al. (2019)
Forecasting and Early Warning	No forecasting tools or early warning systems for rice blast. Farmers rely on visual symptoms, leading to delayed interventions.	China and Japan have advanced forecasting systems that use satellite monitoring and weather data. Bangladesh has piloted ICT-based tools.	Adhikari et al. (2022); Zhu et al., 2021
Genetic Approaches	Limited molecular breeding and slow deployment of resistant varieties.	China, India, and the Philippines use marker-assisted breeding and transgenic techniques. Japan employs pyramiding resistance genes to manage pathogen evolution.	Joshi et al. (2017); Li et al. (2019)

Soils are degraded due to soil erosion and the declining use of FYM or compost, as well as limited integrated nutrient management (NARC, 2021; Paudel et al., 2019). Reduced soil organic carbon, high nitrogen levels, poor soil pH, and inappropriate irrigation management contribute to increased susceptibility to rice blast (NARC, 2021; FAO, 2020). Additionally, low silicon content increases blast disease since silica localises on the leaf surface, acting as a physical barrier against blast fungus penetration (Ishiguro, 2001).

Poor pest and disease management practices, coupled with limited knowledge of integrated pest management (IPM) and reliance on harmful chemical pesticides, exacerbate crop losses (Subedi et al., 2020). While modern practices have boosted yields, they also pose new challenges for managing diseases like rice blast.

The poor rice blast management in Nepal is largely due to the lack of awareness and knowledge on rice disease management among farmers, extension workers, and agricultural stakeholders. Without knowing how to prevent, detect it early, or control it effectively, farmers are not able to take the right decision to manage rice blast. This issue is especially concerning in Nepal’s hilly areas where extension support is weak. Farmers sometimes attribute the disease to weather conditions and resort to burning crops when the blast becomes severe (**Figure 1**).

### Genetic approach for controlling rice blast in Nepal

Rice blast issue in Nepal could be managed through a combination of using resistant varieties with integrated disease management practices. Currently, built-in genetic trait for blast resistance is considered as the most cost-effective, easy to adopt and sustainable method of crop production for the smallholder farmers in low-input agriculture (Ou, 1985; Bonman, 1992; Bonman et al., 1992). It is also economical and environmentally friendly (Khan et al., 2001; Haq et al., 2002). Research by NARC on rice blast through multilocation rice blast screening nurseries known as National Rice Blast Screening Nurseries identified and recommended several blast-resistant cultivars for Nepal (**Figures 2**, **3**; **Supplementary Table 1**). Hundreds of rice genotypes are being tested annually for blast resistance at various agroecological domains in Nepal. Laxmi and Sabitri are established resistant varieties often used as checks in National Rice Blast Screening Nurseries, while Masuli and Sankharika serve as susceptible checks. Studies indicate that blast resistance in varieties like Laxmi is governed by a single dominant gene on chromosome 8 (Sharma et al., 2007). Blast-resistance varieties such as Khumal-1, Khumal-2, Khumal-3, Radha-12, Chandannath-1, Chandannath-3, Sabitri, and Palung-2 offer an economical method for controlling blast. However, these varieties provide only partial resistance to the disease (Bhandari et al., 2017).

Despite numerous recommended cultivars, resistance, especially governed by major genes, can break down earlier under field conditions (Kiyosawa, 1982; Bonman, 1988), leading to significant yield losses. Hence, identifying new sources of resistance particularly partial resistance and developing a suitable deployment strategy is crucial for effective blast management (Sharma et al., 2012). Research in Nepal has identified 15 blast resistance genes Pi-54, Piy2(t), Pi-d(t)1, Pi-z, Pi-a, Pi-k, Pi-y1(t), Pi-44, Pi-b, Pi-g(t), Pi-29, Pi-11, Pi-ta and Pi20(t) among Nepalese rice gene pools (Bhatta et al., 2012). A subsequent review by Joshi et al. (2014b) reported 40 blast resistance genes from several rice landraces with multiple alleles, including both dominant and recessive. Joshi et al. (2014b) suggests utilizing a broad spectrum of resistance genes and pyramiding genes and QTLs for effective blast management in Nepal. In fact, combining or pyramiding multiple resistance genes into one variety boosts broad-spectrum resistance, reducing pathogen adaptation. Recent advancements in understanding the genetic basis of blast resistance, together with closely monitoring the rice blast population structure helped to identify durable blast resistance gene combinations. For example, Yasuda et al. (2015) achieved better suppression of leaf blast when two genes pi21 + Pi35 were combined compared to either pi21, Pi34 or Pi35 individually. At the International Rice Research Institute (IRRI), progress is being made to introgress a combination of Pi9, Pik-h and Pi35 into various elite rice varieties, making them available for rice breeders worldwide.

Using resistant varieties is the most cost-effective and easily adaptable strategy to manage rice blast for farmers, however, a challenge is the development of blast-resistant new rice varieties usually takes over 10 years. Rice breeders in Nepal are working toward the use of modern rice breeding technologies to expedite the development of new rice blast resistant varieties. The use of molecular-assisted breeding in combination with speed breeding could reduce the development of new rice varieties from over 10 years to 5 years (Mba, and Ogbonnaya, 2022). Advances in genomics and molecular techniques have enabled the development of markers linked to specific resistance genes. Techniques like rapid fragment length polymorphism (RFLP), single nucleotide polymorphism (SNP), simple sequence repeat (SSR) and Kompetitive allele-specific PCR (KASP) tightly linked to target genes are widely used molecular-assisted selection (MAS) in rice breeding for developing new rice varieties with a combination of all the traits including blast resistance (Sharma et al., 2012). KASP markers have been used to develop blast resistant Jumli Marshi variety for high hills of Nepal in collaboration with Bangor University, UK. Using both genotypic and phenotypic markers is highly effective for the selection of durable and resistant varieties (Vasudevan et al., 2014). Despite being in its infancy in Nepal, MAS is gaining popularity due to increased access to molecular tools and sequencing facilities.

By manipulating environmental conditions such as light, temperature, and photoperiod, speed breeding creates optimal growth conditions for plants, making it possible to advance up to six segregating generations per year in different crops, including rice and thereby reducing the breeding cycle and seed cycle by half. This approach offers great potential for integrating crop improvement with modern crop breeding technologies, such as genome editing, MAS including KASP and genomic selection for accelerating crop improvement (Watson et al., 2018). In this way, new genetic gains in new rice varieties including blast resistance can be delivered to farmers without delay. This approach enables breeders to respond rapidly to emerging blast pathotypes and speed up the development of blast-resistant rice varieties. With its ability to shorten breeding timelines and increase the rate of genetic gain, speed breeding holds immense promise for the development of climate-resilient rice varieties capable of withstanding blast disease outbreaks, thereby contributing to global food security.

CRISPR-Cas9 technology enables precise genetic modifications, facilitating the development of improved rice varieties with enhanced blast resistance. Researchers have successfully edited genes like OsERF922 to enhance resistance (Wang et al., 2016). This technology is increasingly used to convert susceptible varieties into resistant ones (Wang et al., 2016; Zhou et al., 2022). The use of CRISPR-Cas9 in Nepal has been initiated to improve Jumli Marshi and some other susceptible varieties for blast resistance. These advancements offer promising opportunities for developing durable, high-yielding, and sustainable blast-resistant rice varieties.

### Integrated practices for effective rice blast management

**Figure 6:**
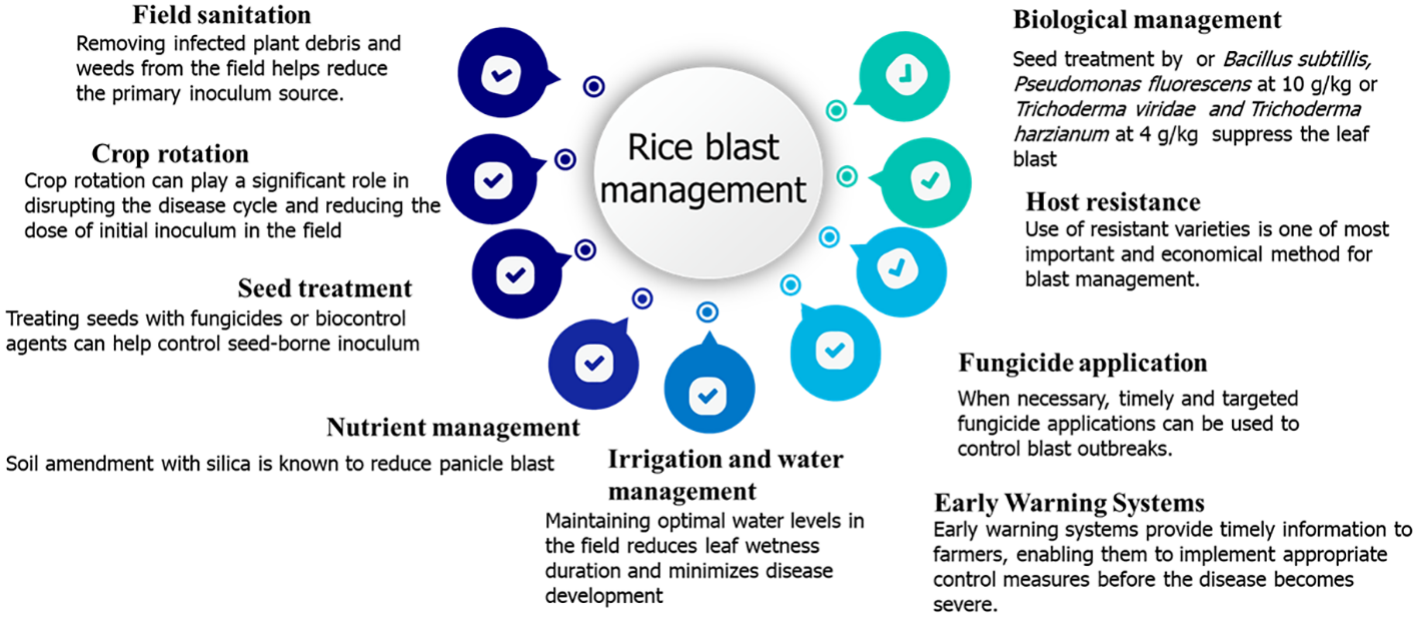
Practice to be adopted for the successful management of rice blast in Nepal.

Integrated agricultural practices that have been evaluated or practiced for the successful management of rice blast in Nepal are outlined in **Figure 6**.

#### Cultivation practices

Cultivation practices from sowing to harvesting are vital for reducing blast epidemics. Early planting disrupts the pathogen’s life cycle, and practices like removing plant debris, long crop rotation, summer ploughing, and clean cultivation or weeding (removal of collateral hosts) in the main field help mitigate blast incidence (Sharma et al., 2007; Sah, 1993). Similarly, maintaining optimal water levels or flooding rice fields creates anaerobic conditions which are unfavourable for pathogens. Raising rice seedlings in wet seedbeds minimises rice leaf blast (Sah, 1989, 1993).

#### Seed treatment

Earlier studies on rice seed sorting with salt solution, e.g. 20% for coarse rice and 15% for fine rice significantly removed diseased and lighter seeds and ultimately contributed to healthy seedling production. Seed treatment with tricyclazole has shown substantial reductions in blast severity (Chaudhary and Sah, 1998), emphasizing the importance of disease-free, high-quality seeds. The practice also improved plant stand establishment in direct-seeded rice (DSR) (Manandhar et al., 2006).

#### Varietal diversification and crop rotation

Continuous planting of susceptible rice varieties in a monoculture can increase blast prevalence (Mustafa et al., 2019) due to the ideal environment it creates for disease spread. Conversely, planting genetically diverse varieties together is an effective ecological strategy for disease management and sustaining crop productivity. For example, the varietal rotation minimised blast epidemics on Jumli Marshi in Nepal’s Jumla valley (3000 m) (Manandhar, 2017).

Crop rotation is effective in disrupting the rice blast disease cycle and reducing inoculum levels in the field. Blast fungus in temperate regions can survive in rice stubble year-round while in the tropical Terai of Nepal “green-bridge” created by multiple rice crops per year are responsible for building the pathogen inoculum in the fields making rotation with non-host crops is essential.

#### Use of bio-control agents and biostimulants for blast management

Options include *Bacillus subtilis* strains B-332, 1Pe2, 2R37 and 1Re14 were found effective in blast suppression (Changqing et al., 2010). Seed treatment by *Pseudomonas fluorescens* at 10 g kg^-1^ or *Trichoderma viride* or *T. harzianum* at 4 g kg^-1^ inhibit rice blast (Yang et al., 2008; Chaudhary et al., 2014; Mukherjee et al., 2010). *Streptomyces* treatment of infected rice seedlings reduced rice blast incidence by 88.3% (Law et al., 2017). Application of avirulent isolates of *P. oryzae* and non-rice pathogen *Bipolaris sorokiniana* reduced rice blast under field conditions (Manandhar et al., 1998a, 2000).

Bio-stimulants offer environmentally friendly alternatives to chemical pesticides. Garlic extract at high doses and neem extract at 4 ml/15 ml PDA medium inhibit the mycelial growth of *P. oryzae* (Khanzada and Shah, 2012). Garlic and its compound allicin have been reported effective against the rice blast fungus (Slusarenko et al., 2008; Fiona et al., 2005). Foliar spray of titepati (*Artemisia vulgaris*) leaf extract reduced leaf blast by 47.6% in field trials (NPPRC, 2019). These integrated management practices provide sustainable solutions with environmental co-benefits, particularly beneficial for farmers in economically challenged regions like Nepal.

#### Managing rice blast using fungicides

When necessary, fungicide applications are employed to control blast outbreaks, but their use must be carefully managed to mitigate environmental impact and prevent fungicide resistance. Various fungicides currently used for managing rice blast in Nepal are listed in **Supplementary Table 2**. Farmers commonly rely upon chemical fungicides like carbendazim (Manandhar, 1984; Manandhar et al., 1985; Sah and Karki, 1989) and tricyclazole (Chaudhary and Sah, 1998; Ghimire et al., 2017) due to their accessibility and rapid efficacy. Applying tricyclazole 22% and hexaconazole 3% SC three times at weekly intervals from the booting stage controlled 87% and 79.6% leaf and neck blast, respectively (Magar et al., 2015). Propiconazole application during tillering and panicle initiation stages demonstrated optimal efficacy in controlling leaf blast (NRRP, 2017; Acharya et al., 2020). Moktan et al. (2021) reported varying degrees of leaf blast control: 61% with tricyclazole 75% WP, 46% with biomycin 3% SC, 36% with propiconazole 25% EC, 35% with Captan 70% + Hexane 5% WP, 31% with hexaconazole 5% SC, and 9% with validamycin 3% L compared to untreated controls. Additionally, Ghimire et al. (2017) noted suppression of neck blast incidences by 75% with tricyclazole, 59% with hexaconazole, and 44% with kasugamycin, compared to non-treated controls. Tirmali et al. (2001) reported tricyclazole to be effective for blast management at maximum tillering, panicle initiation and at the heading stage of the crop.

Continuous use of fungicides with the same group and mode of action increases the risk of developing pathogen resistance. To mitigate this, it is recommended to apply fungicides either in a tank mix or in rotation. Resistance to several fungicides has already been observed in *Magnaporthe oryzae*. For example, melanin biosynthesis inhibitor targeting scytalone dehydratase (MBI-D) fungicides such as carpropamid, diclocymet, and fenoxanil, introduced in Japan in 1998, quickly established as a major fungicides for rice blast management due to their prolonged efficacy, low application rates, and reduced frequency. However, resistance developed within a short period, leading to their discontinuation in Japan by 2003. Similarly, D’Ávila et al. (2021) reported a significant increase in resistance of rice blast pathogens to fungicides from the Quinone outside inhibitor (QoI) group (e.g., azoxystrobin, trifloxystrobin), the melanin biosynthesis inhibitor (MBI) group (e.g., tricyclazole), and the sterol demethylation inhibitor (DMI) group (e.g., tebuconazole) in Brazil. However, tricyclazole, a widely used MBI fungicide for rice blast management in Nepal, is considered to have a low risk of resistance development due to its two distinct target sites involved in melanin biosynthesis (Kurahashi, 2001; D’Ávila et al., 2021). Despite 30 years of continuous use in Japan, no resistance to MBI-R fungicides has been reported in *M. oryzae* (Skamnioti and Gurr, 2009).

Soil drenching or foliar application of non-fungicidal chemicals like ferric chloride, di-potassium phosphate, and salicylic acid have been explored for rice blast management, indicating potential induced resistance (Manandhar et al., 1998a). The blast fungus has developed mechanisms to counteract or evade the rice plant’s immune responses.

### Forecasting of rice blast and adoption of early warning system

Weather conditions significantly influence rice blast development, with weather-based forecasting models can be developed utilizing historical data to predict disease outbreaks. Weather-based forecasting aligned with rice growth stages could be more reliable for blast forecasting. However, forecasting rice blast remains challenging due to the complexity of pathogen-host-environment interactions. The accuracy of models depends on data availability and surveillance effectiveness, with ongoing research improving forecasting techniques. Effective forecasting enables proactive measures, minimizing losses and environmental impact while optimizing crop management. The substantial global use of fungicides for rice blast management could be curtailed through the adoption of blast prediction models. Various methods have been employed to forecast rice blast effects to customize warning systems for different regions (Lanoiselet et al., 2002; Ashizawa et al., 2005) (**Supplementary Table 2**). Commonly used models for blast disease outbreak projection are PYRICULARIA (Gunther, 1986), LEAFBLAST (Torres, 1986), and EPIBLAST (Kim and Kim, 1993). Various computer simulation-based models for rice blast forecasting are now available (**Supplementary Table 3**). However, work on weather-based forecasting of rice blast is at a juvenile stage in Nepal.

### Policy support and stakeholders collaboration in rice blast management

Nepal has policy instruments and stakeholder collaboration frameworks in place to support the management of rice blast, but their effective implementation remains largely inadequate. Key policies directly or indirectly related to the management of rice blast include the Plant Protection Act (2007), which ensures seed certification and quarantine measures to prevent disease spread, the National Seed Policy (1999), which promotes use of disease-resistant rice varieties, and Integrated Pest Management (IPM) policy, which emphasizes sustainable practices, such as use of healthy seed, blast-resistant varieties, balanced plant nutrients, pesticides judiciously etc (MoALD, 2007; ADS, 2015; MoAD, 1999). Climate adaptation plans and fertilizer subsidy policies also indirectly support blast management (NAPA, 2010; MoALD, 2019).

Collaboration among stakeholders remains fragmented. Government institutions, such as the Ministry of Agriculture and Livestock Development and NARC, lead policy formulation and research, while local governments focus on providing extension services. National Rice Research Program (NRRP) develops and popularises resistant rice varieties (NRRP, 2022). All the stakeholders promote IPM strategies. The private sector is critical for distributing seeds of resistant varieties and other inputs, including fungicides. NGOs and international knowledge partners like IRRI provide technical support and capacity building (IRRI, 2020). Farmers, despite being central to implementing management strategies, are often excluded from planning processes.

Despite efforts, implementation failure of policy instruments, weak coordination, limited funding, and inadequate research-extension linkages hinder effective collaboration. Strengthening multi-stakeholder platforms, involving farmers in decision-making, and fostering public-private partnerships are essential to enhance implementation. Training extension agents, promoting awareness among farmers, and incentivizing private-sector participation can ensure sustainable and integrated management of rice blast in Nepal (MoALD, 2015; IRRI, 2020).

Early warning, forecasting, and decision support systems are essential for effective rice blast management. However, developing, validating, and implementing these systems requires substantial investment, long-term commitment, and institutional support. Currently, Nepal lacks the policies and institutional framework needed to develop such systems.

### Global relevance of the research

Rice blast is a devastating disease causing 10–30% yield losses and threatening food security globally, especially in Nepal and other Himalayan regions. The Himalayan foothills are hotspots for blast fungus diversity, where rapidly evolving pathotypes frequently overcome resistance in major rice varieties. Evidence from the recent epidemic of rice panicle blast in Nepal indicates the shifting climatic conditions, particularly during panicle initiation, are intensifying the risk of such outbreaks.

A 40-year review of rice blast research in Nepal reveals rising disease pressure driven by changing weather patterns. Nepal’s genetic diversity in rice and blast pathogens, coupled with its varied agroecological zones, makes it a key location for studying pathogen evolution and disease management, and varietal screening with lessons applicable across the Himalayan region and beyond (**Table 1**). For instance, a Nepalese isolate has been identified as a globally aggressive and virulent strain, highlighting the broader relevance of these findings.

Proposed strategies for sustainable management include developing blast-resistant, climate-resilient rice varieties using molecular tools and speed breeding, forecasting disease and pathotype emergence, and integrating modern fungicides, plant defence activators, and biological controls. Optimizing planting schedules, agronomic practices, and seed systems is equally crucial in Nepal as well in other rice growing regions in the world.

This study underscores the need for regional and international collaboration to address rice blast challenges across the Himalayan belt, offering scalable solutions to enhance food security for smallholder farmers in similar agroecological settings worldwide.

## Recommendation and way forward

Nepal is renowned for its genetic diversity in rice, diverse blast pathogens, varied agroecological zones, and climatic diversity. However, the complex interactions among these elements and their impact on rice blast disease incidence and severity are not well understood. Prioritising research on these interactions is essential for breeding blast-resistant rice varieties as well as for deploying effective disease management strategies in Nepal. Western Nepal has been identified as one of the important biodiversity hotspots for blast fungus and one of the Nepalese blast isolates has been reported as highly virulent and aggressive isolate on the global level.

Nepal’s diversity in blast pathogens, rice varieties, agroecological zones, and climates positions it as an ideal global hub for rice blast phenotyping research. Nepal should initiate international collaboration on this research theme.

Breeding blast-resistant, climate-resilient rice varieties with a combination of essential traits using advanced breeding methods and speed breeding approaches is vital for delivering new genetic gains to farmers’ fields rapidly for ensuring food and nutrition security for smallholder farmers. This approach also supports sustainable and eco-friendly management of blast disease.

Exchanging poor-quality rice seeds either through formal or informal systems, significantly contributes in spreading new races of rice blast pathogens to different regions. Ensuring pathogen-free seeds is crucial for managing rice blast. Prioritising the deployment of new, disease-resistant, and multi-stress-tolerant rice varieties is crucial.

Nepal’s extensive rice cultivation across various terrains and altitudes showcases its unique genetic diversity. However, it remains unclear whether this varietal diversity helps manage rice blast or fosters new pathogen races. Future research should aim to clarify this relationship.

Key strategies for effective rice blast management in Nepal include monitoring pathogen populations, using resistant varieties, ensuring seed quality, promoting sustainable practices and educating farmers can help reduce rice blast impact and maintain food security.

## Data Availability

The original contributions presented in the study are included in the article/**Supplementary Material**. Further inquiries can be directed to the corresponding author.

## Supplementary material

Supplementary Figure 1 Analysis of relative humidity data of rice season for the last 50 years (1980-2023) in central hills of Nepal, data recorded at Khumaltar, Lalitpur.

Supplementary Figure 2 Analysis of minimum temperature of rice season for last 50 years (1980-2023) in central hills of Nepal, data recorded at Khumaltar, Lalitpur.

Supplementary Figure 3 Analysis of daily rainfall data of rice season for the last 50 years (1980-2023) in central hills of Nepal, data recorded at Khumaltar, Lalitpur.
