# Supplementary figures and images for: Defending rice crop from blast disease in the context of climate change for food security in Nepal
Source: Front Plant Sci. 2025 Jun 25;16:1511945. doi: 10.3389/fpls.2025.1511945 (PMC12238087; doi:10.3389/fpls.2025.1511945)

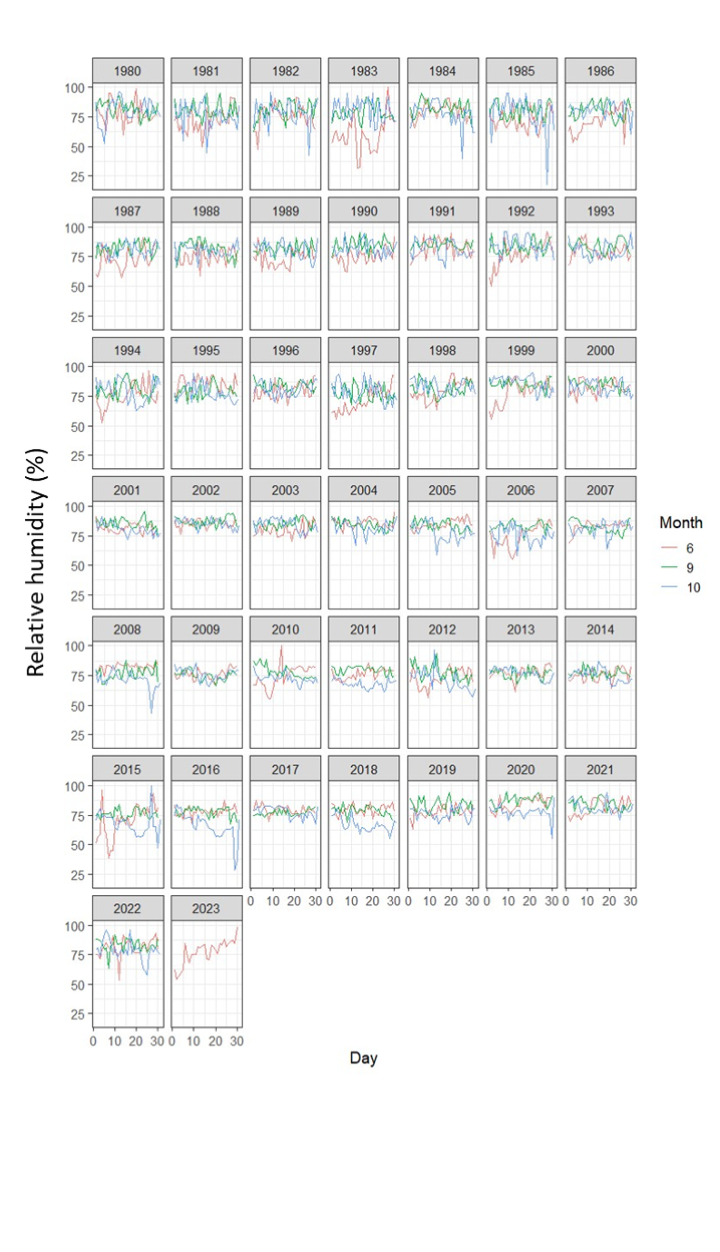

Supplement: Supplementary Figure 1 — Analysis of relative humidity data of rice season for the last 50 years (1980-2023) in central hills of Nepal, data recorded at Khumaltar, Lalitpur. [file Image1.tiff]

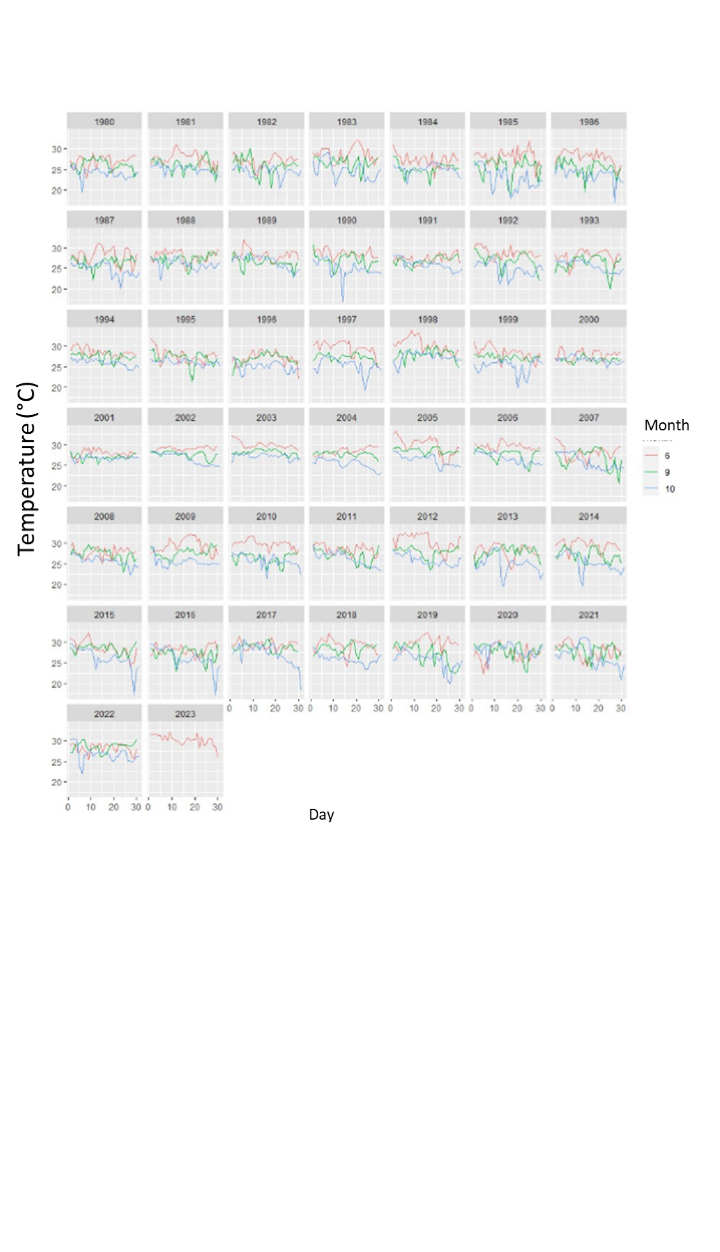

Supplement: Supplementary Figure 2 — Analysis of minimum temperature of rice season for last 50 years (1980-2023) in central hills of Nepal, data recorded at Khumaltar, Lalitpur. [file Image2.tiff]

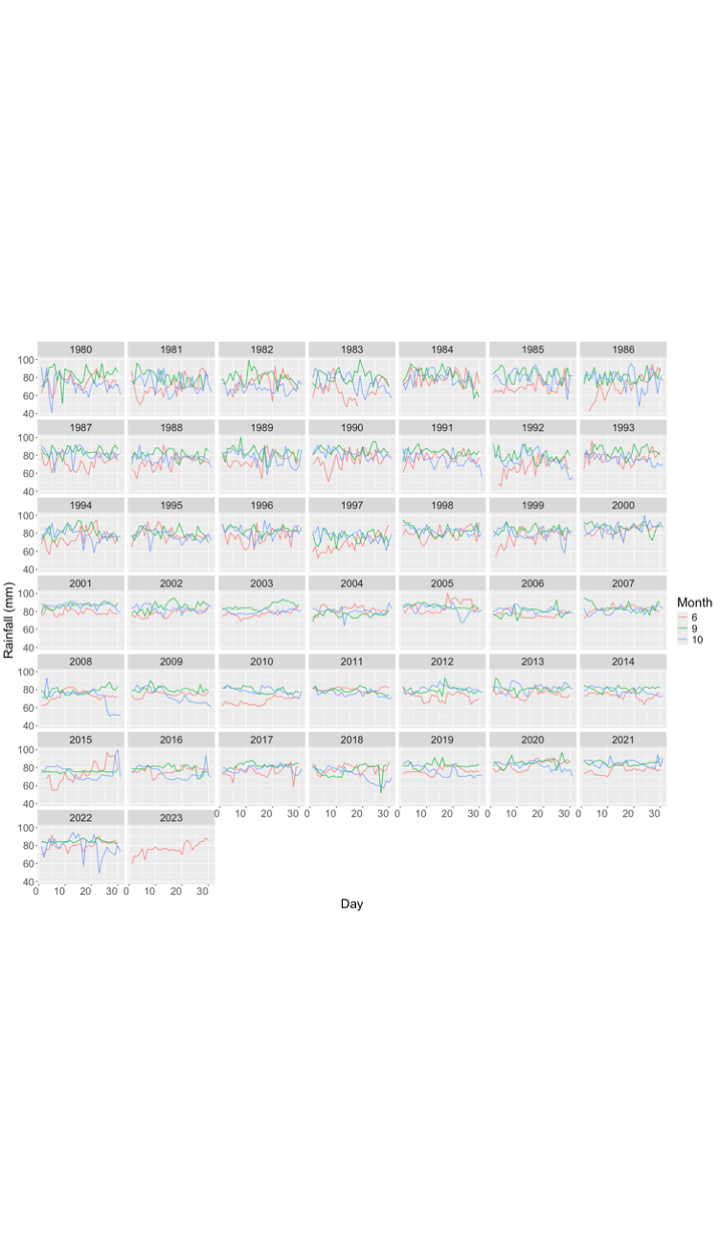

Supplement: Supplementary Figure 3 — Analysis of daily rainfall data of rice season for the last 50 years (1980-2023) in central hills of Nepal, data recorded at Khumaltar, Lalitpur. [file Image3.tiff]
