# Supplementary material for: Defending rice crop from blast disease in the context of climate change for food security in Nepal
Source: Front Plant Sci. 2025 Jun 25;16:1511945. doi: 10.3389/fpls.2025.1511945 (PMC12238087; doi:10.3389/fpls.2025.1511945)
Supplement: Supplementary file 4 [file Table1.docx]

**Supplementary Tables**

**Supplementary Table 1**. Some of the rice genotypes resistant with neck and leaf blast in blast screening nurseries tested at Khumaltar, Lalitpur and Gokarna, Kathmandu

| **Year of Testing** | **Neck blast^x^** | **Leaf blast^y^** |
| --- | --- | --- |
| 2019-20 |  | TP 30578, IR 106522-39-37-1-1-B-B-5, NR 2175-66-2-3-1-1, NR 2208-14-3-2-2-1,IR 17L-1544, IR 15L-1505, IR 15L-1505, SVIN072 |
| 2020-21 | NR 10676-B-5-3, NR11130-B-B-B-19-3, NR11137-B-B-B-10, NR 11570-B-B-5 and NGRC 01795 | NR10676-B-5-3, NR 11115-B-B-31-3, NR 11105-B-B-27, NR 11271-B-B-6, IR 88965-39-1-6-4, NR 11321-B-B-7-3, NR 11105-B-B-20-2-1, Manjushree2, Black fine rice, Purple Rice, Chandnnath-3,NR10838-B-B-4, PR29399-B-2-2-1, Lekali Dhan-1, Lekali dhan-3,NR11366-B-B-6-1-2 |
| 2021-22 |  | SVIN255,SVIN257, Sabitri |
| 2022-23 | NR 10676-B-5-3, Black fine rice, NR 11130-B-B-B-19-3, NR 11379-B-B-22-3, NR 11476-B-B-10-1,NR 11443-B-B-7, NR 11443-B-B-10-1, NR 11475-B-B-9-3, NR 11581-B-B-3, NR 11607-B-B-11, NR 11571-B-B-5, NR11645-B-B-8, NR11647-B-B-10, NR11647-B-B-13, NR11648-B-B-12, NR11649-B-B-9, NR11653-B-B-6, NR11658-B-B-2, NR11661-B-B-11, NR11662-B-B-7, NR11666-B-B-3, NR11666-B-B-6, NR11674-B-B-18, NR11683-B-B-13, NR11686-B-B-4, NR11686-B-B-11, NR11701-B-B-3, NR11366-B-B-6-1-2, NR 11415-B-B-7-1, NR11648-B-B-12, NR11658-B-B-2 genotypes were found resistant | IR 16A3708,IR 17L 1430, NR 2188-8-2-1-2-1, IR 16A3025, SVIN 052,IR 16F 1014, Tarahara-107,Tarahara-109, Tarahara-2, NR 2188-43-1-2-2-5-1, Sukkhadhan-3, Radha 4, NR 2286-13-2-1-1-3, Chaite-5, IR 13F 1284, NR2188-43-1-2-11, Hardinath Boro dhan-1, Ghaiya-3, Hardinath-6, IR15D1024, Gangasagar, Sunaulo Sugandha, HardinathBoroDhan-1,  NR 10676-B-5-3, NR 11377-B-B-22-1, Black fine rice, NR 11585-B-B-18, Khumal-4, NR 11440-B-B-14, NR 11139-B-B-B-13-2, NR 11544-B-B-3, NR 11477-B-B-2-2, NR 11374-B-B-17-2, NR 11572-B-B-6, NR 11452-B-B-8, NR 11381-B-B-17-2, NR 11577-B-B-11,IR13K190,NR 11345-B-B-15-1-2PR29399-B-2-2-1, NR 11341-B-B-32-2,NR10838-B-B-4, NR11366-B-B-6-1-2-1-1, NR11345-B-B-2-3-2, NR 11345-B-B-2-3-2-1-3, NR 11586-B-B-15, NR 11321-B-B-7-2-2, NR 11475-B-B-9-2,NR 11568-B-B-5, NR 11515-B-B-1, NR 11443-B-B-10-1 |

^x^Standard Neck blast screening nursery was established and neck blast scoring was done at (20-25 days after heading using 0-9 scale (IRRI, 2002) and neck blast severity was calculated and genotypes were categorized as resistant and susceptible.

^y^Experiments were performed in field conditions under standard blast screening nursery protocol developed by IRRI (IRRI 2002), resistant (Sabitri or Laxmi) and susceptible (Shankarika or Mansuli) checks were planted in between every 20 entries to monitor the disease pressure, scoring was done using IRRI leaf blast scoring scale and genotypes were categorized.

**Supplementary Table 2**: Fungicides used for rice blast management in Nepal

| **Fungicide** | **Mode** | **Reference** |
| --- | --- | --- |
| Edifenphos (Hinosan) | Folier spray | (Thapa 1975 and Manandhar *et al* 1985) |
| Kasumin | Soil drenching | (Thapa 1975 and Manandhar *et a*l 1985) |
| Iprobenfos (Kitazin) | Folier spray | (Thapa 1983, Manandhar *et al* 1985) |
| Probenazole (Oryzemate ®) | Folier spray | (Thapa 1983, Manandhar *et al* 1985) |
| Benomyl (Benlate) | Folier spray | (Upadhyay 1983) |
| Topsin M (topsin) | Folier spray | (Manandhar *et al* 1987b) |
| Carbendazim (Bavistin) | Folier spray | (Manandhar 1984,  and Sah and Karki 1988) |
| Tricyclazole (Beam) | Folier spray | (Chaudhary and Sah 1998) |
| Tricyclazole 22% +  hexaconazole 3% SC | Folier spray | (Magar et al 2015) |
| Ferric chloride | Folier spray | (Manandhar *et al* 1998a),  Manandhar *et al* 2000) |
| Di-potassium phosphate  and salicylic acid | Folier spray | (Manandhar *et al* 1998a),  (Manandhar *et al* 2000) |
| Non-rice pathogen  *Bipolaris sorokiniana* | Folier spray | (Manandhar *et al* 1998b) |
| *Trichoderma harzianum* | Folier spray | (Chaudhary *et al* 2014) |

**Supplementary Table** 3. List of different forecasting models used to predict blast disease (Leaf and Neck).

| **Forecasting Models** | **First Developed** | **References** |
| --- | --- | --- |
| BLASTCAST | Japan | (Ohta *et al.*, 1982) |
| PYTRICULARIA | Netherland | (Gunther, 1986) |
| Leaf blast simulation model | Philippines | (Torres, 1986) |
| PYRNEW | Indonesia | (Tastra *et al.*, 1987b) |
| EPIBLA | India | (Manibhushanrao & Krishnan, 1991) |
| EPIBLAST | Korea | (Kim & Kim, 1993) |
| DYMEX and CLIMEX | Australia | (Lanoiselet *et al.*, 2002) |
| BLASTMUL | Japan | (Ashizawa *et al.*, 2005) |
| Machine learning technique | India | (Kaundal *et al.*, 2006) |
| EPIRICE | Korea | (Savary *et al.*, 2012) |
